# Challenges to implementing national comprehensive sexuality education curricula in low- and middle-income countries: Case studies of Ghana, Kenya, Peru and Guatemala

**DOI:** 10.1371/journal.pone.0200513

**Published:** 2018-07-11

**Authors:** Sarah C. Keogh, Melissa Stillman, Kofi Awusabo-Asare, Estelle Sidze, Ana Silvia Monzón, Angélica Motta, Ellie Leong

**Affiliations:** 1 Guttmacher Institute, New York, United States of America; 2 Department of Population and Health, University of Cape Coast, Cape Coast, Ghana; 3 African Population and Health Research Center, Nairobi, Kenya; 4 Facultad Latinoamericana de Ciencias Sociales, Ciudad de Guatemala, Guatemala; 5 Unidad de Salud, Sexualidad y Desarrollo Humano, Universidad Peruana Cayetano Heredia, Miraflores-Lima, Peru; Aga Khan University, KENYA

## Abstract

School-based comprehensive sexuality education (CSE) can help adolescents achieve their full potential and realize their sexual and reproductive health and rights. This is particularly pressing in low- and middle-income countries (LMICs), where high rates of unintended pregnancy and STIs among adolescents can limit countries’ ability to capitalize on the demographic dividend. While many LMICs have developed CSE curricula, their full implementation is often hindered by challenges around program planning and roll-out at the national and local level. A better understanding of these barriers, and similarities and differences across countries, can help devise strategies to improve implementation; yet few studies have examined these barriers. This paper analyzes the challenges to the implementation of national CSE curricula in four LMICs: Ghana, Kenya, Peru and Guatemala. It presents qualitative findings from in-depth interviews with central and local government officials, civil society representatives, and community level stakeholders ranging from religious leaders to youth representatives. Qualitative findings are complemented by quantitative results from surveys of principals, teachers who teach CSE topics, and students aged 15–17 in a representative sample of 60–80 secondary schools distributed across three regions in each country, for a total of around 3000 students per country. Challenges encountered were strikingly similar across countries. Program planning-related challenges included insufficient and piecemeal funding for CSE; lack of coordination of the various efforts by central and local government, NGOs and development partners; and inadequate systems for monitoring and evaluating teachers and students on CSE. Curriculum implementation-related challenges included inadequate weight given to CSE when integrated into other subjects, insufficient adaptation of the curriculum to local contexts, and limited stakeholder participation in curriculum development. While challenges were similar across countries, the strategies used to overcome them were different, and offer useful lessons to improve implementation for these and other low- and middle-income countries facing similar challenges.

## Introduction

Young people aged 10 to 24 years account for just over a quarter of the population in low- and middle-income countries (LMICs) [1], and constitute the human capital of these countries [2]. Ensuring young people achieve their full potential by providing them with education and skills at this stage in the evolving knowledge-intensive economy will help to capitalize on the demographic dividend and improve the developmental trajectory of LMICs. Yet many young people in these countries lack the information they need to be better prepared to prevent adverse sexual and reproductive health outcomes such as early marriage, sexual coercion and violence, unintended pregnancy, unsafe abortion, and sexually transmitted infections including HIV [3, 4]. In these settings, school-based comprehensive sexuality education (CSE) can help empower adolescents with the knowledge and practical skills to make safe and informed choices with regards to their sexual and reproductive lives [5–10].

In recent years, many LMICs have developed CSE policies and context-specific curricula. However, the translation of these policies and curricula from paper into practice is not always straightforward, and a range of bottlenecks can hinder their full implementation in schools. These bottlenecks can operate at various levels, from national program planning to the classroom level. There have been relatively few studies of the implementation of national CSE programs in LMICs [11–13], and even fewer specifically examining challenges to implementation [12, 13]. A better understanding of the major challenges encountered in the process of implementation can help devise strategies to address them. In particular, analyzing barriers from a range of different settings can highlight commonalities and differences and help to draw out lessons that may be applicable beyond these particular settings. The cross-country evidence that does exist suggests that implementation in LMICs is often slow, but there is little investigation into the factors responsible for this [14, 15]. This paper provides an analysis of the challenges to implementation of national CSE curricula in secondary schools in four LMICs: Ghana, Kenya, Peru and Guatemala.

These four countries represent a range of contexts with respect to adolescent sexual and reproductive health (ASRH) issues and the programs in place to address them. The proportion of 15–19 year-olds who have initiated sex ranges from 28% in Guatemala [16] to 39% in Kenya [17], and few of those sexually active are using contraception, with modern method prevalence as low as 22% in Ghana [18] and 31% in Guatemala [16]. This puts adolescents at risk of unintended pregnancy: the majority of births to 15–19 year-olds (calculated for the five years preceding the surveys) were unplanned, from 59% in Kenya [17] to 74% in Ghana [18]. Many other unintended pregnancies end in unsafe abortion [18–21]. Each country has also faced particular challenges: for example, Kenya, and Ghana to a lesser extent, has had to tackle HIV infection in its young population [22, 23], while Guatemala and Peru have seen particularly high rates of sexual violence against adolescents [24–27]. To address these challenges, these four governments have developed strategies to address ASRH issues that, although varying in scope and level of implementation, include some form of school-based CSE.

While the scope of sexuality education should be understood within a global framework that has evolved over time, countries’ educational settings and cultural contexts differ and must be reconciled with the values and priorities inherent in international definitions and guidelines [7, 8, 28]. In this paper, we define CSE as education on sexual and reproductive health and rights that provides a structured opportunity for adolescents to gain knowledge and practical life skills necessary for making healthy informed choices about their sexual and reproductive lives. For sexuality education to be comprehensive in the range of topics offered, it should cover five core areas: sexual and reproductive physiology; contraception and unintended pregnancy; HIV/STI prevention; gender and sexual and reproductive rights; and values and interpersonal skills. Since the focus of this paper is on uncovering challenges to implementation of the CSE curricula (rather than on evaluating their comprehensiveness, which is examined elsewhere [29–32]), for simplicity, we use the term CSE for all four countries, regardless of whether the curriculum is truly comprehensive or not.

The first building block necessary for a successful CSE program is an enabling environment, which encompasses positive cultural norms and values, infrastructure needs (such as youth-friendly and youth-responsive SRH services, supportive media, and links between schools and the community), as well as strong policy-level and community-level support [33, 34]. This enabling environment precedes implementation, and is therefore beyond the scope of this paper. This paper focuses on challenges to implementation from the national program planning level to roll-out at the local (district) level. It does not analyze challenges faced at the school or classroom level, which are extensive enough to warrant a separate examination. While teacher training is a key element of program implementation, the quality of teacher training and associated challenges are best evaluated in relation to teachers’ performance in the classroom, and are therefore best discussed in the context of school-level barriers. Since curriculum development mostly occurs prior to program implementation, the paper will not provide a detailed analysis of the curriculum development process. However, it will touch on certain aspects that contribute to implementation success, such as young people’s involvement in curriculum development, and the adaptation of curricula to local contexts.

## Methodology

### Data collection

This paper is based primarily on key informant interviews conducted in four countries in 2015. The countries were selected from two regions (Latin America and Africa), with one country from each region that is relatively more advanced in its CSE implementation (Peru and Ghana), and one country at a relatively earlier stage of implementation (Guatemala and Kenya), based on reviews of policy and other documents [35–37] and personal communication with researchers and other stakeholders in the regions. In order to be selected, a country had to have established CSE policies and curricula, and some political support for their implementation.

Three regions were selected for the study in each country to represent geographic, ethnic and cultural diversity, and included the capital region where central government is located:

- Ghana: Greater Accra, Brong Ahafo, Northern
- Kenya: Nairobi, Homabay, Mombasa
- Peru: Lima, Ayacucho, Ucayali
- Guatemala: Guatemala City, Huehuetenango, Chiquimula

In each country, 20 to 30 in-depth interviews were conducted with key informants. These included policy makers, curriculum developers, program planners and implementers from central, regional and local government, as well as NGO and international agency representatives, advocacy groups, community leaders, youth organizations, parent-teacher association representatives and religious leaders at the national level and local level in the selected regions. A breakdown of informants by category is given in Table 1. Key informants from government, international agencies, NGOs and other advocacy groups were identified by the research team based on their role in the organization and approached directly about participating in the study. Informants at the community level were identified with the help of community leaders, village chiefs, NGOs working at the community level, or other individuals knowledgeable about adolescent sexual and reproductive health in the communities in which they live.

**Table 1:** Key informants interviewed, by type and country.

	Kenya	Ghana	Peru	Guatemala
Policy-makers	1	1	2	3
Curriculum developers	1	1	1	1
Program implementers (national or local government)	6	3	10	5
International cooperation	1	1	2	1
National civil society organizations, advocates	9	2	4	4
Youth organizations	4	3	5	2
Community-based organizations (other than youth)	3	5	3	5
Religious and traditional leaders	2	4	3	4
**TOTAL**	**27**	**20**	**30**	**25**

Informants were asked about their experiences with the CSE program, opinions about major challenges and opportunities, and suggestions for improvement. A semi-structured interview guide was used to ensure that all key topics were covered. Interviews were conducted in English (in Ghana and Kenya, except for a few community-level interviews in Kiswahili in Kenya) and Spanish (in Peru and Guatemala) by experienced qualitative researchers trained on the interview guide during a three-day workshop. Interviews were audio-recorded subject to the informant’s informed consent, transcribed, and imported into NVivo qualitative software [38] for coding. The few interviews in Kiswahili were transcribed and translated into English immediately after interview. Spanish transcripts were coded and analyzed directly in Spanish and quotes used in this paper were translated by the authors at the time of writing.

This paper also reports briefly on results from surveys conducted with principals, CSE teachers and students aged 15–17 in a representative random sample of secondary schools from the selected regions in each of the focus countries in 2015 (Table 2). The surveys were conducted concurrently with the in-depth interviews. The aim of the surveys was to examine how the curriculum was implemented in schools. The sample of secondary schools was drawn so as to be representative of each of the three selected regions in each country, stratified by public/private and girls-only/boys-only/co-educational. All respondents gave their informed consent prior to being surveyed. Trained interviewers interviewed school principals and 2–5 teachers per school (depending on school size) who taught CSE topics in their classes. The principal survey asked about how CSE was delivered in their school, monitoring and evaluation systems, and support for CSE teachers. The teacher survey asked teachers what CSE topics they covered, what teaching methods they used, what training they had received, and how teachers and students were assessed on CSE teaching and learning. Students aged 15–17 were invited to fill in a self-administered questionnaire; researchers were available to answer any questions during the completion process. In small schools, all students 15–17 were invited to participate, while in larger schools students were randomly selected for participation. For students in Guatemala, Ghana and Kenya, the school principal acts as guardian during school hours, so consent was sought from the principal for the students to be surveyed. In Peru, informed consent had to be sought and obtained from parents prior to the survey. Student surveys asked about what CSE topics they had learned, and their perceptions and opinions of the CSE curriculum.

**Table 2:** Sample sizes for secondary school surveys.

	Kenya	Ghana	Peru	Guatemala
Number of schools surveyed	78	82	61	80
Number of principals surveyed	73	78	57	80
Number of teachers surveyed	196	346	210	188
Numbers of students surveyed	2484	2990	2528	3004

This paper presents results only from the monitoring and evaluation component of the three surveys, since (although the data are collected at the school level) monitoring and evaluation systems are a key component of national-level implementation processes. The remainder of the survey results will be the subject of a separate paper looking at classroom-level implementation challenges.

The Guttmacher Institute partnered with a local research organization in each country to conduct the study: University of Cape Coast (Ghana), the African Population and Health Research Centre (Kenya), Universidad Peruana Cayetano Heredia (Peru), and Facultad Latinoamericana de Ciencias Sociales (Guatemala). Ethical approval was obtained from the University of Cape Coast Institutional Review Board in Ghana, AMREF-ESRC in Kenya, the Comité Institucional de Ética para Humanos of the Universidad Peruana Cayetano Heredia in Peru, as well as the Guttmacher Institutional Review Board. No separate IRB approval was necessary for Guatemala.

### Data analysis

The in-depth interviews were analyzed in NVivo [38] using thematic analysis. Interviews were coded into pre-determined high-level nodes based on the themes of interest around challenges to implementation–for example “dealing with opposition”, “monitoring and evaluation”, etc. These codes were kept broad so as to avoid overly compartmentalizing the data and to remain open to emerging themes. The high-level nodes were then further analyzed and coded to uncover specific barriers to program implementation within these broad categories, with no a priori coding scheme. Survey data were double-entered into CSPro [39] and exported to STATA14 [40] for analysis. The results presented here are descriptive statistics, and are used here to quantify some of the challenges described in the in-depth interviews. The results section is structured around the major challenges encountered by the government and other stakeholders in their efforts to implement the CSE curriculum at the national and local levels.

## Results

### The integrated model of CSE

In most LMICs including the four case study countries, CSE is not offered as a standalone subject in school, but is integrated into other subjects. Key informants from all countries mentioned benefits to this: integration demonstrates how CSE is related to other subjects and thereby permeates all aspects of life; integration also allows space for the teaching of CSE without adding another subject into overcrowded curricula. However, informants pointed out several drawbacks to an integrated approach: teachers trained in their main subject areas are rarely taught how to integrate CSE, and they can more easily skip over topics they consider controversial, with the excuse that they do not have adequate knowledge to cover them. Integration can diminish the importance of CSE in the curriculum, as it gets diluted into other subjects and does not wield the weight of a standalone subject for teachers or students:

For it as standalone, one of the good things you gain is the details that you go through, and being looked at as a subject and given the priority that it deserves, so you end up achieving the targets of your objectives. When it is integrated, probably as any other component…for example put it within biology, it is crowded and the message can also be clouded, so you don’t go into the detail. [International agency representative, Nairobi, Kenya]

Moreover, many of the key subjects into which CSE is integrated are elective and/or non-examinable. This means that some students miss out on the teachings because they do not elect to take those particular classes, or, if the subject is not examinable, less attention may be paid to it given the pressure on teachers and students to focus on topics that will be examined. In Peru, CSE topics are taught in *tutoria*, an hourly personal development class, but CSE is reportedly frequently neglected in favor of other topics like drugs. In Ghana, Management in Living, the curriculum with arguably the most comprehensive range of CSE topics, is an elective which is taken mostly by girls, evidencing an enduring perception among some Ministry of Education (MoE) officials that CSE is more relevant to girls:

You realize that there are certain subjects, you say it’s for females. Like Management in Living, you see. I think they were looking at it that way because the reproduction part is with the females, so these are the things they should know more about. This is what my personal view is. So even if you say a boy in the second cycle should go and do Home Economics [of which Management in Living is a part], he doesn’t want to do it. Even if he will do, he will not do the Management in Living. Unless it becomes compulsory for everyone, the boys will not opt for it. […] It is like, Home Economics is for women and because of that, let us give them this knowledge. [MoE official, Accra, Ghana]

However, in both Peru and Ghana, some CSE content is integrated into examinable subjects, thus ensuring that students’ knowledge of at least some topics is assessed. In Kenya, where CSE is mostly taught as part of the compulsory but non-examinable Life Skills curriculum (which teachers often skip to focus on core examinable subjects, according to several informants), a MoE representative suggested that moving CSE into examinable subjects could encourage teachers to pay attention to it:

If you bring in health education as a subject per se, and it is non-examinable, it will die immediately so it is better you teach it infused in the examinable, because if you just say this is non-examinable, that will be the death of that subject. Nobody will teach things without a mean score. [MoE official, Homabay, Kenya]

The view that examination encourages both teachers and students to dedicate more time to CSE was echoed by informants from the other countries. However, some key informants pointed out that while integrating CSE into other subjects and examining them through these subjects works well for knowledge-based topics, it is less obvious how well it works for values and skills, where the emphasis on passing examinations is not always helpful.

Various solutions to these problems were proposed. In order to ensure that all students receive comprehensive information, it might be useful to complement these integrated portions, particularly in elective subjects, with a dedicated subject that is examinable, even if less weight is assigned to it compared to other examinable subjects. This would enable teachers to cover CSE in more depth and better effect change:

My first approach will be that the snippets of integrated SRH from various subjects, aggregate [them] together into an SRH course that one expects that you should do and pass and get some national accreditation for it. That will motivate the students and that will also motivate the teachers to be able to teach it. But for now it is not like that, you don’t receive any reward to show that you have gone through the course, all we know is that you have passed your biology, you have passed your chemistry, and you have passed your physics. You can pass a biology exam without answering one question on sexuality and reproductive health. And even the sexual and reproductive health issues that are integrated into that, is basically the anatomy. [Development partner, Accra, Ghana]

In Kenya it was suggested that the existing Life Skills curriculum could be developed into a standalone examinable CSE subject. But others involved in curriculum development and implementation warned that creating a standalone subject would require that teachers be trained specifically in that subject.

### Challenges in program coordination

Informants from all four countries explained how the centralized education system helps to ensure that CSE content is standardized across the country, facilitating national cohesion. However, in some cases centralization can create challenges in implementation, as regional directorates need to obtain permission from the central government in order to implement CSE initiatives:

The curriculum on sexual and reproductive health has been taken to one level then to the other, and they have been told “no, go and amend this and you come back and start from here”… You know, it takes lot of time. At least if it was decentralized, I think it would be better. When it comes to maybe issues of giving them the go ahead to do it, the Ministry should be involved, but the others, they should at least decentralize to avoid this red tape kind of system that tends to delay everything. [Secondary School Heads Association, Kenya]

In some of the countries, central government guidelines do not always filter down to the local level, partly due to differing regional priorities. For example in Peru, the *Education Guidelines* have not been implemented by local education authorities, mainly because they prefer to allocate their limited funds to higher profile issues such as malnutrition, and because there is no dedicated CSE team to monitor the implementation. This has led to a lack of coordination, as individual schools try to implement initiatives as best they can, often with the help of NGOs:

These guidelines are on paper, there is no good strategy to implement them in practice. Sometimes they train us, they train health professionals on these topics, but this is not accompanied by funding. So you train them but you don’t give them the materials to do the workshops, you don’t give them the necessary resources to convene the workshops. There are many professionals who are trained, they are convinced [of the value of CSE], they are sensitized and they do teach it, with support from a few NGOs, or they develop their own materials themselves… But there is no financing chain. […] In the budget for each region there is like 3500 soles, nothing more, to dedicate to CSE. [Ministry of Health official, Ucayali, Peru]

Lack of coordination between central government, local government and NGO efforts is also a challenge in the other countries, and leads to confusion over whose mandate it is to implement CSE in schools: in the survey, 48–56% of principals (depending on the country) stated that it was the government’s mandate, while the rest believed it was the responsibility of the school or (to a lesser extent) the teachers themselves (data not shown). Centralization also restricts potential and much-needed funds for CSE. For example in Ghana, parent-teacher associations are ready to contribute money to improve CSE in their schools, but schools cannot take the funds without central government approval.

Several informants from different countries emphasized the importance of having a dedicated program for CSE with an assigned budget within the MoE, even—or especially—if it is not being taught as a standalone subject. This could help coordinate efforts across the different subject areas as well as between central and local government and NGOs, to ensure continuity and avoid duplication of efforts. As a Peruvian MoE official put it:

The Ministry of Education needs a specific unit that is responsible for all the CSE processes. Because otherwise, it doesn’t work. CSE has always been an additional obligation for someone. Or in the additional budget of someone. It really needs to have a unit or program that takes responsibility for all the processes. [MoE official, Lima, Peru]

Such a program exists in Ghana in the form of the government-run School Health Education Program (SHEP), which coordinates all CSE content taught in schools through both the official curriculum and co-curricular activities. Although it also allows NGOs and international agencies to run their own programs in certain districts, there is little overall coordination of efforts between the various programs. In Peru, the 2008 *Education Guidelines and Pedagogical Orientations for CSE* set out a model for teaching CSE competencies across the curriculum, with a dedicated team within the *tutoria* directorate (DITOE) in the MoE, but due to changes in government (including the dissolution of DITOE), implementation has remained weak. According to a government informant, having a dedicated permanent team independent of changes in government is crucial for progress. In Kenya and Guatemala, CSE is not coordinated through a separate program, and implementation in these countries tends to be weaker overall.

### Curriculum adaptation

The “one-size-fits-all” curricular approach of the centralized system, while providing the benefit of standardization, can limit the potential relevance of CSE to local contexts:

“[A centralized system] will deliver the national objectives in education but not really a context-specific education. Like for instance when you look at issues that you might need to address, assuming you want to address issues in West Pokot or Kajiado, you might want to have content which is FGM and GBV [gender-based violence] specific, but we are saying issues might be irrelevant in Makueni or some other areas where they don’t practice FGM. You might not want to use the same methodology to talk about HIV, or even the same emphasis when you are doing it in Kisumu, such areas with high HIV prevalence compared to some other areas which are considered to have low prevalence rates.” [NGO representative, Nairobi, Kenya]

While local education authorities may have autonomy in allocating budgets to CSE (as in Ghana under SHEP), they may not always be involved in developing the curriculum, so cannot ensure the inclusion of locally relevant content. Nonetheless, teachers in all four countries are encouraged by central and local authorities to adapt the curriculum to meet the specific needs of their students, based on the local context. However, not all teachers are sufficiently equipped or comfortable with the material to be able to adapt it to their settings:

These materials need to be appropriate for… For example, if they were distributed here in Pucallpa City, they could be used without any inconvenience, but if they are distributed in rural or indigenous areas, then there would be an issue of contextualization, which would require that the teachers work to adapt the materials. And in reality, this is what doesn’t happen. The teachers do not manage to do it, they themselves comment that not all of them have mastered the methodology to do the adaptation, the contextualization. [NGO representative, Ucayali, Peru]

Therefore, there is a need for official adaptations of the curriculum to different cultural contexts, in terms of content as well as language in some countries. In countries like Ghana and Kenya with a multitude of local languages, it is not expected or feasible that the curriculum be translated into all languages. However, in countries like Peru dominated by a few major languages (Spanish, Quechua, Aymara), official translation into these languages can facilitate curriculum adoption. Several key informants in Guatemala and Peru stressed the importance of making curricula and materials available in local languages. Although some teaching on HIV has been adapted from the official curriculum to the needs of different regions in Guatemala, and some CSE content has reportedly been adapted to rural areas in Peru, overall, culturally specific materials are lacking. As a Guatemalan Ministry of Health representative puts it:

This is a deficiency of the National Curriculum, which is, let’s say, generalized and standardized for a country that has considerable diversity. Even Huehuetenango has nine Mayan ethnic groups, so each one with their own customs, traditions, their own culture. I think it would be really good if each region could adapt the curriculum according to their daily reality. [Ministry of Health official, Huehuetenango, Guatemala]

### Stakeholder participation in curriculum development

The inclusion of young people in curriculum development is also key to ensuring the content is adequately tailored to their needs. Moreover, including young people at an early stage may encourage their engagement with the material and enhance their views of themselves as agents of their own change [6, 8]. According to a Ghanaian youth representative:

“There should be increased and meaningful engagement of young people in all these processes, because this essentially is about young people and so they should be involved actively throughout these processes. More importantly, we should move beyond making commitments and putting these things in documents. We should take those concrete steps that will ensure that we have better health outcomes for young people. Their health is not just beneficial to them as young people, it is equally essential for the nation in its quest for development.” [Youth-focused NGO representative, Accra, Ghana]

In Ghana, young people reported that although they are consulted on policy issues and curriculum development, their views on curriculum development are not sufficiently taken into consideration. Similarly, in Peru, informants complained that young people’s voices are collected by the government, but are rarely incorporated into CSE guidelines or other documents. In Guatemala, curriculum design is also top-down, taking little account of the perspectives and needs of the student population. Kenya’s curriculum review is perhaps the most consultative, drawing on input from a wide range of stakeholders, including adolescents, gathered together through public meetings throughout the country:

“[We involve] all the major groups in Kenya… and then we have the NGOs… now the various education NGOs, we have health NGOs involved in that, the civil society organizations, and we try to have a few adolescents represented—last time we got a few, actually that is what I missed on, there are a few who were represented. Adolescents and adolescent organizations.” [Ministry of Health, Nairobi, Kenya]

However, despite the benefits of consultations with various stakeholders, there may also be drawbacks. It is difficult to reach a consensus on certain sexual and reproductive health issues that are considered sensitive. For example, depending on the country, discussion on the inclusion of topics such as contraceptives, safe abortion, sexual orientation and gender identity can be protracted. In addition, in all countries, opposition from certain quarters, such as religious groups, can stall CSE curriculum development or implementation processes, especially when such groups wield power close to (or even within) the government, as is the case in these countries:

I think that opposition from a few stakeholders within the Ministry of Education is negatively impacting the strategy, stalling its progress. [National NGO representative, Guatemala City, Guatemala]

Parental opposition (stemming from concerns that CSE encourages sexual activity) was found to be strong in certain countries, and it has reportedly slowed CSE implementation in some schools in Guatemala. This opposition appears to be coming from a small but vocal minority, since in the survey, 90–95% of students (depending on the country) reported that their parents supported CSE. Yet students’ perceptions differed from those of their teachers: the proportions of teachers who reported parental support were lower, at 67% in Kenya, 69% in Ghana, 45% in Peru and 38% in Guatemala. To tackle parental opposition, all four countries have government and NGO initiatives sensitizing parents to the importance of CSE, for example through *escuelas de padres* (parent schools) in Guatemala and Peru:

“There are parent schools in all secondary schools. All the tutoria teachers have to help run the parent school. Let’s say, I have a class—first year of secondary, section A let’s say—so I’m a tutoria teacher for these students. So I have to see how my boys, my girls are doing, what are their needs, what difficulties they have. And to address those difficulties, those needs, I call the parents. [Local education authority, Ayacucho, Peru]

### Funding issues

The lack of dedicated funding for CSE from governments has posed a challenge for program implementation. Historically, funding for CSE has been piecemeal, mainly from external sources, and tied to specific projects. This manifests itself in a patchwork of localized programs run by international agencies, which often cannot be sustained by the government when agency funding ends:

“Over the years, there have been many institutions that have come and run programs. But only for a period. The time comes when their project ends, so they leave and nothing remains. And this has continued to happen again and again. So there needs to be more sustainability. If a program comes, then the regional government or the regional authorities need to take responsibility for it, so that it continues. Because it’s in this continuity that we will find success. CSE is ongoing work, no? Because we don’t change people from one day to the next; the change is slow, it is incremental. [Local education authority official, Ucayali, Peru]

With government CSE funding tight in all four countries, governments have welcomed NGOs and international donors to roll out local CSE initiatives. For example, Kenya has a host of different government-approved curricula being implemented by NGOs around the country [41]. Peru has also had various agency programs running over the years, which upon withdrawal, have left trained teachers unable to implement what they learnt for lack of government funding. In Ghana, a government official observed:

“It is normally tied with donor funding. You know, government is not spending on adolescent reproductive health programs. So it is the donors who are supporting in those areas. And they have focused areas. So unless they give you funding, you are limited.” [MoE official, Accra, Ghana]

This multiplicity of donors, while filling an important gap where government funding is lacking, creates discontinuity in program delivery and a lack of standardization in the information students receive, as funders have different priority areas and are not coordinating with each other. In Guatemala, in contrast, recent CSE initiatives have largely been led by UNFPA through pilot programs distributing manuals and training teachers and technical staff, but progress has stalled due to lack of government support [29]. According to an international agency representative:

“What the Ministry of Education’s DIGECUR [curriculum development unit] wanted was that [CSE] classes cover how “you can get pregnant like this, you can’t get pregnant like that, this is how you menstruate”, and that’s it. “HIV is transmitted this way.” They don’t say anymore, you don’t talk about decision-making, you don’t talk about anything that has to do with transforming this world and the people’s history, nothing about human rights, nothing about this or that. [International agency representative, Guatemala City, Guatemala]

Guatemala’s current conservative government is much less supportive of CSE than the previous government, and has considerably slowed implementation. The effect of government changes on support for and prioritization of CSE over the years can also be seen in Peru, where guidelines have been reworked with successive governments, thus slowing implementation.

### Monitoring and evaluation

Strong monitoring and evaluation is another key ingredient for a successful program. In all countries, government bodies are in charge of monitoring the performance of teachers. The survey revealed some amount of confusion over who should be monitoring the teaching of CSE in schools in the four countries, with some school principals claiming that the government was responsible, others claiming that the school itself was responsible, and some attributing responsibility to both entities (Table 3). A non-negligible proportion of principals (7–15%, and up to 40% in Guatemala) believed that no-one was responsible.

**Table 3:** Monitoring and evaluation in schools.

	Kenya	Ghana	Peru	Guatemala
**ACCORDING TO PRINCIPALS (all schools)**	(N = 77)	(N = 78)	(N = 57)	(N = 80)
Entity responsible for M&E of CSE teaching				
School level	67.8	80.2	71.7	34.5
Government	60.2	37.2	24.2	27.3
No one	7.3	14.9	9.2	39.7
**ACCORDING TO TEACHERS (all schools)**	(N = 196)	(N = 346)	(N = 210)	(N = 188)
Student assessments of CSE learning				
External exams	48.6	85.1	na	na
End of year/term exams	78.1	99.2	31.3	7.2
Continuous assessments	69.6	93.8	30.8	74.8
No assessment	13.2	0.0	35.7	18.2
**Among teachers reporting students are assessed**	(N = 168)	(N = 343)	(N = 127)	(N = 141)
Method of student assessment				
Oral assessment	37.5	66.1	36.2	31.9
Written exam/test	97.6	98.0	48.2	85.6
Projects	7.7	27.2	46.0	41.4
Practical demonstrations	7.9	21.8	18.5	18.2
Presentations	21.3	30.1	41.8	48.2
Group work	22.3	41.7	36.1	29.3
Aspect of student learning assessed				
Knowledge	91.1	97.8	72.8	88.1
Attitudes	58.3	57.7	61.5	70.9
Practical/life skills	48.4	53.3	36.1	34.3
**ACCORDING TO STUDENTS (all schools)**	(N = 2,484)	(N = 2,990)	(N = 2,528)	(N = 3,004)
Are CSE topics included in exams?	76.0	98.7	57.1	44.4

While monitoring the quality and comprehensiveness of teaching on CSE generally falls under the purview of regional or local authorities, this monitoring may be done infrequently or inconsistently within and across schools, and mechanisms for reporting back to the central level are often weak. As CSE is integrated into other subjects, it is particularly difficult to monitor: informants explained that given the infrequency of monitoring activities, it is unlikely that an inspector will monitor a class specifically at the time that they happen to be teaching CSE content. Moreover, evaluations are performed on the teaching as a whole, not on specific subject components. As such, no specific and consistent monitoring of CSE is being conducted in these countries:

We are not monitoring as much as we should. So we cannot vouch for every school, every teacher that they are actually teaching all the issues about adolescent reproductive health. So that is another challenge. The feedback mechanism for us to know how much is being taught is not adequate. [MoE official, Accra, Ghana]

The CSE workshops offered in schools by the health sector, with support from international agencies, are more closely evaluated, but even this evaluation consists more in confirming that the workshops happened than in collecting any meaningful impact measures. Key informants in Kenya and Ghana also noted the lack of impact evaluations to determine the effect of CSE on student outcomes. One Peruvian informant emphasized the key role of such evaluations in program success:

Interviewer: And from the political level, how do you think CSE could be given more support?

Respondent: First, it would have to be budgeted. It would need to be regulated. And most of all, include a budget for monitoring and follow-up. And there should be an evaluation: before starting, half-way through and at four, five years, to measure if we are truly succeeding in having an impact on those young people. [NGO representative, Ucayali, Peru]

Students were, however, evaluated on some aspects of their CSE learning. In the surveys, in Ghana and Kenya, the majority of teachers reported that CSE was part of end-of-year examinations and continuous assessments (Table 3). Yet in Peru only 31% of teachers reported CSE was part of each of these, while in Guatemala CSE was often included in continuous assessments (75%) but not in end-of-year examinations (7%). Students confirmed that CSE was included in examinations in Ghana (99%) and Kenya (76%), and students were more likely than teachers to report it was included in Peru (57%) and Guatemala (44%). In Peru, 36% of teachers reported that there was no student assessment on CSE. While the majority of evaluations were written tests, some teachers also reported using other assessment methods such as projects, presentations and group work. The teachers reported evaluating students mostly on knowledge (50–98%), and less on attitudes (43–71%) and skills (24–53%).

## Discussion

The analysis of key informant interviews and survey data uncovered several challenges hindering implementation of CSE in these four countries. This paper’s focus on challenges does not diminish the countries’ achievements in the field of CSE: the four countries were selected because of their established CSE policies and curricula, and some degree of political support for their implementation. The paper seeks to highlight areas where national and local planning and implementation can be improved, and to draw out lessons learned for other LMICs.

The centralized education system in Ghana, Kenya, Peru and Guatemala can be an asset in terms of oversight and standardization of CSE curricula, but centralization can also cause unnecessary bureaucracy, as budget allocation decisions are sometimes delayed, guidelines do not always filter down to the local level, and accountability for implementation is elusive. The piecemeal funding situation further complicates an already complex landscape, as financially strapped governments rely on international donors and NGOs to roll out localized CSE programs, but are not coordinating the different efforts in time, space and content, and cannot take over and ensure continuity when the donor or NGO withdraws. Disjointed and unpredictable funding is a challenge that afflicts CSE programs in many resource-limited settings [12, 13, 42].

Having a dedicated permanent team at the central and regional level (independent of government changes) with an assigned budget in the MoE could enable greater coordination of NGO and donor activities and thereby better coverage and continuity of programs within each country. Ghana’s School Health Education Program (SHEP) effectively coordinates government CSE efforts, but is less involved in coordinating initiatives from other agencies. A dedicated CSE team can also facilitate coordination between the local and central level in areas such as budget allocation and monitoring and evaluation. Where appropriate, budgetary decisions on CSE should be devolved to the local level to ensure a more efficient use of time and resources–a strategy that has been implemented to a certain extent by all countries, at least on paper. Countries who have a dedicated CSE team or program, such as Ghana, and previously Peru, tend to have stronger, more coordinated CSE implementation.

Additionally, having a dedicated CSE team or program can encourage governments to prioritize CSE in budget allocation. While this would not resolve the issue of insufficient funding available overall for education in these resource-constrained settings, it can influence the distribution of available funds. In Ghana, SHEP has helped channel limited education funds towards health and CSE. Sensitizing central and regional education authorities to the important role CSE plays in social and economic development may encourage greater investment in it. Governments’ lack of prioritization of CSE in the face of competing demands on available resources has been shown to be a challenge to implementation in other LMICs [13], and emerged as an important bottleneck particularly in Guatemala and Peru, where it was fueled in part by opposition from religious groups. Cultural and religious opposition is a common barrier to CSE that can slow implementation in many conservative LMICs [34, 43]. However, the key informant interviews and surveys showed that religious as well as parental opposition in fact both come from a small, albeit vocal, minority in these countries. Successful programs do need buy-in from conservative religious and traditional leaders (Rosen et al 2004), but governments should also be responsive to the needs of young people as well as to the overwhelmingly favorable public opinion on CSE. While most parents supported CSE in schools, they should be further sensitized and educated on CSE [43], for example through “parent schools” as in Peru and Guatemala.

In terms of monitoring and evaluation, a dedicated CSE team at both the central and regional levels could facilitate the creation and implementation of CSE-specific indicators to monitor teachers. Currently, CSE is only evaluated as part of other subjects, and there is no mechanism to ensure it is systematically monitored. For monitoring and evaluation systems to be effectively implemented, including in remote areas, local education authorities need to be incentivized to prioritize CSE through clear guidelines matched with adequate budget allocations and robust systems for reporting back to the central level. These reporting systems should include mechanisms for feedback on implementation hurdles encountered across the country, and provisions for periodically revising the program or curricula based on this feedback.

Pending the implementation of more robust CSE monitoring mechanisms, the best strategy to ensure that CSE is taught is to make it examinable in its entirety–if not as a standalone subject, then as part of other subjects. While examinations can only ensure the acquisition of knowledge, and not the adoption of skills [44], they nonetheless provide a useful benchmark to assess the implementation of CSE. Evidence shows that teachers and students take subjects more seriously when they are examined [8]. In recognition of this, one of the commitments made by the 21 East and Southern African countries as part of the 2013 ESA Ministerial Commitment on CSE was to ensure CSE is examinable [45], and many countries including Malawi, Rwanda, Zambia, Zimbabwe and Lesotho have since followed through by making CSE topics in the curriculum examinable [15]. Although in the four countries studied in this paper some CSE topics are included in student assessments, the scope of evaluation should be expanded to include all topics and give more weight to assessing values and skills where possible. Ensuring that all of CSE is examinable will encourage teachers and students to accord it more time and attention in overcrowded curricula, where the integrated model can cause CSE to get diluted or omitted (particularly as its sensitivity makes it prone to getting neglected in favor of other topics), and teachers are often not trained specifically on CSE.

Another challenge of centralized systems is ensuring that they effectively take into account differing priorities at the regional and local level. The one-size-fits-all curriculum that is predominantly used in the four countries limits local relevance in terms of culture and language. Although some modifications have been made for specific populations, informants voiced the need for more culturally relevant local adaptations. Curriculum development should draw on a wider range of stakeholders, at both local and national levels, as for example in Kenya. This should include more consultation with young people (the prime beneficiaries of these programs), whose needs can also be diagnosed through local-level needs assessments [33, 43]. The importance of adapting curricula and materials to local contexts has been pointed out in other LMICs with diverse populations [12, 43, 46]. Given teachers’ documented lack of confidence in adapting curricula to their settings and requests for additional guidance, which have also been reported in other settings [47, 48], there is a need for both national and regional involvement in adapting curricula. Centrally-led curriculum adaptation to major regions and languages needs to be accompanied by more specific adaptation by regional or local education authorities. The need for teaching to be responsive to local needs emphasizes the importance of teacher monitoring and evaluation being partially devolved to local authorities cognizant of the needs of their communities and able to evaluate teachers’ performance in relation to these needs.

Some limitations to the study are worth highlighting. The qualitative and quantitative studies were conducted in three regions in each country. While the survey results are representative of these three regions, they are not nationally representative. However, by purposively sampling three geographically, ethnically and culturally different areas, we have endeavored to represent the diversity of each country. Since the selected regions included the capital city, the qualitative interviews included not only regional-level and community-level informants, but also staff from the central government, and we believe that the experiences and opinions reported in this paper provide an accurate representation of the views of national-level decision-makers involved in the CSE implementation process, as well as providing snapshots of local-level experiences in each of the regions. In fact, one of the strengths of the sampling design is its ability to bring together the views and experiences of a range of stakeholders from the national to the local level, to build a coherent picture of implementation successes and challenges that would not be possible with a more homogenous sample of informants. The cross-country comparisons offer further insights into how to address these challenges that a one-country study might not have uncovered.

## Conclusion

This paper has offered an analysis of the various challenges encountered by countries in the process of implementing a national CSE curriculum. While certain challenges are more prominent in some settings than others, the commonalities across countries are striking. However, the strategies each country has used to address these barriers are different. This analysis has sought to draw out lessons learned in each setting in order to expand the toolbox of strategies for effective CSE implementation to be used by other countries facing similar challenges, and ensure that adolescents can fully realize their sexual and reproductive health and rights.
